# Enhancing risk communication and environmental crisis management through satellite imagery and AI for air quality index estimation

**DOI:** 10.1016/j.mex.2024.102611

**Published:** 2024-02-10

**Authors:** Kulsawasd Jitkajornwanich, Nattadet Vijaranakul, Saichon Jaiyen, Panu Srestasathiern, Siam Lawawirojwong

**Affiliations:** a College of Media and Communication, Texas Tech University, Lubbock, TX 79409, USA; b Department of Computer Science, School of Science, King Mongkut's Institute of Technology Ladkrabang (KMITL), Bangkok 10520, Thailand; c School of Information Technology, King Mongkut's University of Technology Thonburi (KMUTT), Bangkok 10140, Thailand; d Geo-Informatics and Space Technology Development Agency, GISTDA (Public Organization), Bangkok 10210, Thailand

**Keywords:** Air Quality Index (AQI), Satellite images, Landsat 8, Hybrid supervised machine learning techniques, Regression machine learning techniques, Risk Communication, Environmental Crisis Management, Artificial Intelligence, Air Quality Index Estimation Based on Landsat 8 Images Using Hybrid Supervised Machine Learning Models

## Abstract

Due to climate change, the air pollution problem has become more and more prominent [23]. Air pollution has impacts on people globally, and is considered one of the leading risk factors for premature death worldwide; it was ranked as number 4 according to the website [24]. A study, ‘The Global Burden of Disease,’ reported 4,506,193 deaths were caused by outdoor air pollution in 2019 [22,25]. The air pollution problem is become even more apparent when it comes to developing countries [22], including Thailand, which is considered one of the developing countries [26]. In this research, we focus and analyze the air pollution in Thailand, which has the annual average PM2.5 (particulate matter 2.5) concentration falls in between 15 and 25, classified as the interim target 2 by 2021′s WHO AQG (World Health Organization's Air Quality Guidelines) [27]. (The interim targets refer to areas where the air pollutants concentration is high, with 1 being the highest concentration and decreasing down to 4 [27,28]). However, the methodology proposed here can also be adopted in other areas as well.

During the winter in Thailand, Bangkok and its surrounding metroplex have been facing the issue of air pollution (e.g., PM2.5) every year. Currently, air quality measurement is done by simply implementing physical air quality measurement devices at designated—but limited number of locations. In this work, we propose a method that allows us to estimate the Air Quality Index (AQI) on a larger scale by utilizing Landsat 8 images with machine learning techniques. We propose and compare hybrid models with pure regression models to enhance AQI prediction

based on satellite images. Our hybrid model consists of two parts as follows:

• The classification part and the estimation part, whereas the pure regressor model consists of only one part, which is a pure regression model for AQI estimation.
• The two parts of the hybrid model work hand in hand such that the classification part classifies data points into each class of air quality standard, which is then passed to the estimation part to estimate the final AQI.

From our experiments, after considering all factors and comparing their performances, we conclude that the hybrid model has a slightly better performance than the pure regressor model, although both models can achieve a generally minimum R^2^ (R^2^ > 0.7). We also introduced and tested an additional factor, DOY (day of year), and incorporated it into our model. Additional experiments with similar approaches are also performed and compared. And, the results also show that our hybrid model outperform them.

Keywords: climate change, air pollution, air quality assessment, air quality index, AQI, machine learning, AI, Landsat 8, satellite imagery analysis, environmental data analysis, natural disaster monitoring and management, crisis and disaster management and communication.

## Graphical abstract

**Figure:**
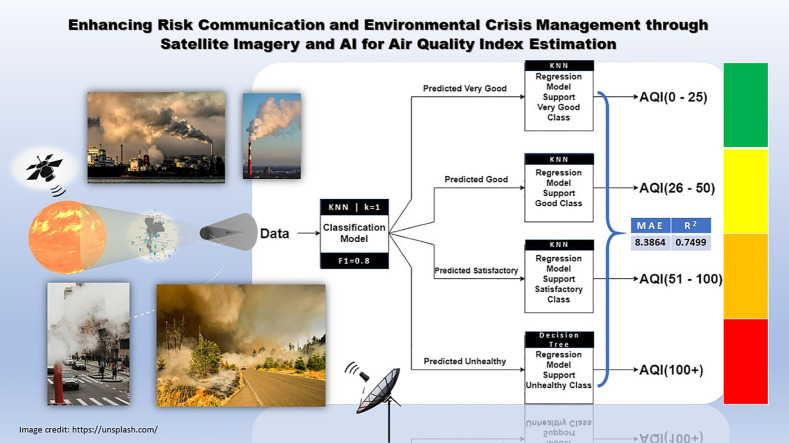

Specifications table

**Table:** 

Subject area:	Environmental Science
More specific subject area:	Crisis Communication and Disaster Management
Name of your method:	Air Quality Index Estimation Based on Landsat 8 Images Using Hybrid Supervised Machine Learning Models
Name and reference of original method:	N.A.
Resource availability:	Landsat 8 images from USGS EarthExplorer, Open Data Cube software, PostgreSQL databases with spatial libraries, Python 3, Python editors such as Google Colab, Python data science libraries such as scikit-learn and pandas, and ground truth data for AQI from Pollution Control Department of Thailand.

## Introduction

### Background

Due to climate change, the problem of air pollution has become increasingly prominent [1]. Air pollution has global impacts on people, and is considered one of the leading risk factors for early death worldwide; it was ranked as number 4 according to the website [2]. One study, ‘The Global Burden of Disease,’ reported 4,506,193 deaths were caused by outdoor air pollution in 2019 [3,4]. The air pollution problem is become even more apparent when it comes to developing countries [3], including Thailand, classified as one of these developing countries [5]. In this research, we focus and analyze the air pollution in Thailand, in which the annual average PM2.5 (particulate matter 2.5) concentration ranges between 15 and 25, classified as the interim target 2 by 2021′s WHO AQG (World Health Organization's Air Quality Guidelines [6]). (The interim targets refer to areas where the air pollutants concentration is high, with 1 being the highest concentration and decreasing down to 4 [6,7]). However, the methodology proposed here can also be adopted in other areas as well.

During the winter in Thailand (usually from November to January) Bangkok and its surrounding metroplex face air pollution problems (including NO2, PM2.5). At present, air quality measurements are conducted by using physical air quality measurement devices at designated but limited locations. Currently, there are approximately 22 air quality measurement stations in and around Bangkok and the Metroplex. The exact radius as to how far each station can cover is unknown, as this information has not been provided by the Pollution Control Department (PCD) of Thailand.

In this work, we propose a methodology in which the spatial data, covering all the study areas and beyond, is derived from satellite images and used in conjunction with machine learning to predict the Air Quality Index (AQI). The satellite imagery used in this work is obtained from Landsat 8, an Earth observation satellite operated by U.S. Geological Survey - USGS and NASA. Landsat 8 re-captures images at the same location every 16 days. The Landsat 8 images used in this work are scene numbers 129050 and 129051 that cover our study area. And, we utilize machine learning techniques to predict AQI values in accordance with the PCD of Thailand's standard.

The dataset used in this work are Landsat 8 Spectral Bands, Vegetation Index (VI), Normalized Difference Vegetation Index (NDVI), Transformed Vegetation Index (TVI), as well as ground truth data that were collected from PCD, matched with the date and time of Landsat 8 Images. Please also note that we used only low cloud confident dataset—otherwise there will be a lot of outliers. The machine learning techniques used in this work are supervised machine learning techniques. We use a hybrid model that combines classification techniques and regression techniques in predicting the AQI values, and compare it a pure regression model. The classification techniques will classify data points into classes according to the PCD of Thailand's standard, whereas the regression techniques estimate the actual AQI value for each class.

Our contributions in this work can be summarized as follows: first, we propose two different techniques – a hybrid machine learning model and a pure regressor model. Second, we compare both techniques in terms of different metrics to determine which one perform well with our dataset. Third, we re-experiment with more newly added datasets and double-check the accuracy, as well as introduce and test a potential factor (DOY: day of year) that might plays an important role in estimating AQI values. Fourth, we perform additional experiments and compare the results with similar approaches.

### Literature review

In 2012 Mozumder et al. [8] proposed a method for air quality assessment using IRS and Landsat 7 data with VI, NDVI and TVI using Linear Regression techniques. In this work, there is no any other machine learning techniques proposed to predict AQI nor Landsat 8, which is newer, was utilized. In 2016, Di et al. [9] proposed an approach for PM2.5 prediction using Aerosol Optical Depth (AOD) data and other spatial data using Neural Networks. In 2017, Pannu et al. [10] used Particle SWARM Optimization (PSO) techniques to predict Benzene - one of air pollution particles. In 2019, Zamani Joharestani et al. [11] used spatial data collected in Tehran, Iran to predict PM2.5 by using machine learning techniques: Random Forest, Extreme Gradient Boosting, and Deep Learning. Sethi and Mittal [12] predicted air quality of Faridabad, India by using AOD data with several machine learning techniques. Wang et al. [13] estimated PM2.5 in China by using AOD data using Neural Networks. In 2020, Sun et al. [14] used MODIS data – one of the prevalent satellite remote sensing data with other spatial data to monitor haze pollution in Shanghai, China. Leong et al. [15] used Support Vector Machine – SVM to predict Air Pollution Index – API (another name of AQI). In 2022, Gu et al. [16] used Hybrid interpretable Artificial Neural Network to predict air pollution. Lin et al. [17] used Convolution Neural Networks for visualizing transboundary air pollution from Himawari-8 satellite images. Ji et al. [18] adapted Weibo social media data as health sensing mechanism to predict and/or corrected estimation of air quality assessment. Saez and Barcelo [19] used a hierarchical Bayesian spatiotemporal method to predict PM10 (a type of air pollution particles) in Catalonia, Spain. We summarize the advantages and disadvantages, along with other key details (i.e., datasets, methods, and study areas) of each paper mentioned here in Table 15.

Having said that, none of the aforementioned papers use Landsat 8 images, whose technologies is mostly similar to the current Landsat 9 [20] (with some updates: [21]), considered the best technology available [22]–but several machine learning models were introduced and experimented. Furthermore, in other domains, we found that machine learning models applied do not necessarily need to be a single model for the entire process, as in Boateng's paper—in that Boateng et al. [23] used both techniques (classification techniques and regression techniques, referred to as hybrid in our paper) to detect syrup adulteration in honey. This, in fact, inspired us to apply these techniques in our work.

### Contribution statements

In this research, we utilize Landsat 8 satellite data, which is widely accepted and freely available, in conjunction with supervised machine learning models to classify air quality data points into the classes of Thailand's PCD air quality standards. Subsequently, a specific model is developed for each of the classes to estimate the final AQI values. Throughout our research, we can summarize the contributions and novelty of our study as follows:

1. By utilizing satellite images and machine learning techniques, we can expand and improve the air quality assessment in a larger scale, especially areas where the physical measurement devices are limited.
2. We utilize the promising technologies of Landsat 8 images, including 11 bands of data, 16 days of repeat cycles, and a spatial resolution as small as 15, 30 and 100 m.
3. We introduce and test with experiments with an additional potential factor (DOY) to incorporate into our model for predicting air quality index classes and values.
4. In this phase of study, we have acquired more datasets, investigated the data as to what types of error exist in our datasets, and performed re-experiments to confirm the accuracy of our model's classification of the previous phase [24].
5. As a continuation from the previous phase, we continue with the estimation of the final AQI values by proposing and comparing the performances of two approaches: a hybrid model approach and a pure regression model approach.
6. We also conduct additional experiments with approaches similar to ours, and compare the results.
7. Finally, for those interested in applying our work in the future, we have detailed our methodology and workflow, as well as the hyperparameters of each model, to ensure that interested parties can use and reproduce our research.

## Materials and methods

In this section, we describe our data sources, the types of data used in our research, ground truth data, as well as the machine learning techniques employed in our research.

### Data sources

Landsat 8 [25] is an Earth observation satellite operated by the United States Geological Survey (USGS) and NASA. Landsat 8 has a spatial resolution as small as 30 m for bands 1 – 7 and 9. The datasets from Landsat 8 used in this work are Level 2 images, including 7 surface reflectance bands (out of the 11 bands) as highlighted in Table 1. Data used in this work must be low cloud confident data to avoid outlier problem. Cloud confident data can be calculated from Pixel_QA data from satellite images itself. Not only do we use the data provided by Table 1, we also incorporate additional data called, Spectral Vegetation Indices [[26], [27]], i.e. Vegetation Index (VI), Normalized Difference Vegetation Index (NDVI), and Transformed Vegetation Index (TVI), into our model in estimating air quality. Spectral vegetation indices can be calculated from other bands using following equations.

1. Vegetation Index (VI)
  (1) VI=NIR−Red
2. Normalized Difference Vegetation Index (NDVI)
  (2) $$NDVI=\frac{NIR-Red}{NIR+Red}$$
3. Transformed Vegetation Index (TVI)
  (3) $$TVI=\;\sqrt{NDVI+0.5}$$

**Table 1:** Surface reflectance spectral bands of Landsat 8.

Band	Name	Wavelength (µm)
1	Coastal Aerosol	0.43 – 0.45
2	Blue (Visible Band)	0.45 – 0.51
3	Green (Visible Band)	0.53 – 0.59
4	Red (Visible Band)	0.64 – 0.67
5	Near Infrared (NIR)	0.85 – 0.88
6	Shortwave Infrared (SWIR) 1	1.57 – 1.65
7	Shortwave Infrared (SWIR) 2	2.11 – 2.29
8	Panchromatic	0.50 - 0.68
9	Cirrus	1.36 – 1.38
10	Thermal Infrared (TIRS) 1	10.6 – 11.19
11	Thermal Infrared (TIRS) 2	11.50 – 12.51

### Ground truth data

**Fig. 1:**
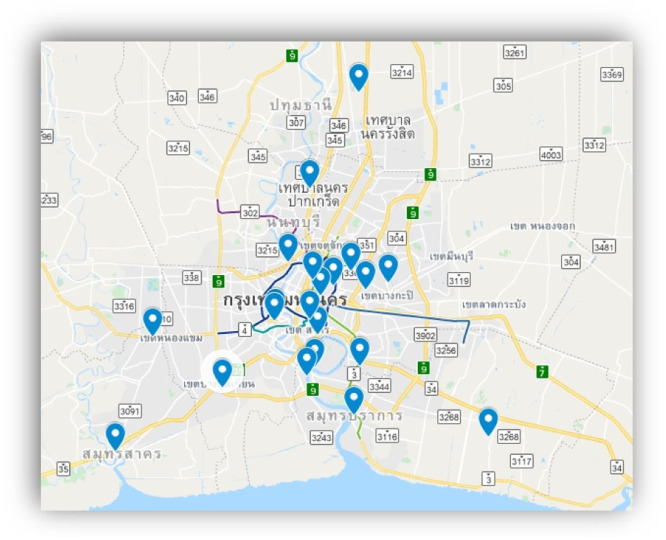
Air Quality measure stations around Bangkok and its Metroplex.

Ground truth data (Air Quality Index Labelled Data [28]) used were collected from Pollution Control Department (PCD) of Thailand and also from www.air4thai.com (official PCD website reporting daily AQI data). These data are collected from actual air quality measure stations around Bangkok and Metroplex, which has about 22 stations (see map Fig. 1). Air quality data can be categorized into 5 classes as shown in Table 2.

**Table 2:** Air Quality in PCD Thailand standard.

Air Quality Index (AQI) (Average of the last 24 h)	Class Value (Quality Standard Class)
0 - 25	Very Good
26 - 50	Good
51 - 100	Satisfactory
101 - 200	Unhealthy (can effect vulnerable people)
201+	Very Unhealthy

### Supervised machine learning techniques

Supervised machine learning is a type of machine learning techniques such that the models learn (are trained) by mapping inputs (datasets with n-features) to desired outputs (actual/labelled values). Two main types of supervised machine learning techniques are classification and regression. Classification models are to classify inputs into the correct classes whereas regression models are to process inputs in order to estimate the actual values. In this work, we experiment with both classification and regression of the supervised machine learning techniques. The supervised machine learning techniques used in this work are decision tree (DT), k-nearest neighbors (KNN), random forest (RF), gradient boosting (GB) and linear regression. Following is a brief description as to what and how each of the supervised machine learning techniques used in this work.

1. Decision Tree (DT) (Used in both classification and regression parts)
  Decision tree is one of the supervised machine learning. Decision tree model is made of data structure called trees, in which internal node and paths are decisive paths and leaf nodes are prediction values.
2. K-Nearest Neighbors (KNN) (Used in both classification and regression)
  KNN is one of the supervised machine learning. KNN does prediction by considering nearest data values using a distance function that can be adjusted depending on how many data points (neighbors or “k”) needed to consider for predictions.
3. Random Forest (RF) (Used in both classification and regression) [29]
  Random forest is one of ensemble machine learning techniques in bagging techniques. Random forest is made from many decision trees combined. Each decision tree has self-structure and self-prediction. The predicted value of the model is from the voting of prediction values among all the trees.
4. Gradient Boosting (GB) (Used in both classification and regression) [30]
  Gradient boosting is one of ensemble machine learning in boosting techniques. Gradient boosting is made of many models of machine learning (sometimes called weak learners) in that each weak learner will learn from previous weak learner and so on.
5. Linear Regression (Used only in regression part)
  Linear regression is a model based on linear equation (y=ax+b) when x is each feature in dataset. Linear regression learns by tuning a coefficient of each feature.

## Model design and evaluations

In this section, we first show the model design of our proposed methods and then demonstrate how the models will be analyzed and evaluated using performance metrics.

### Model design

**Fig. 2:**
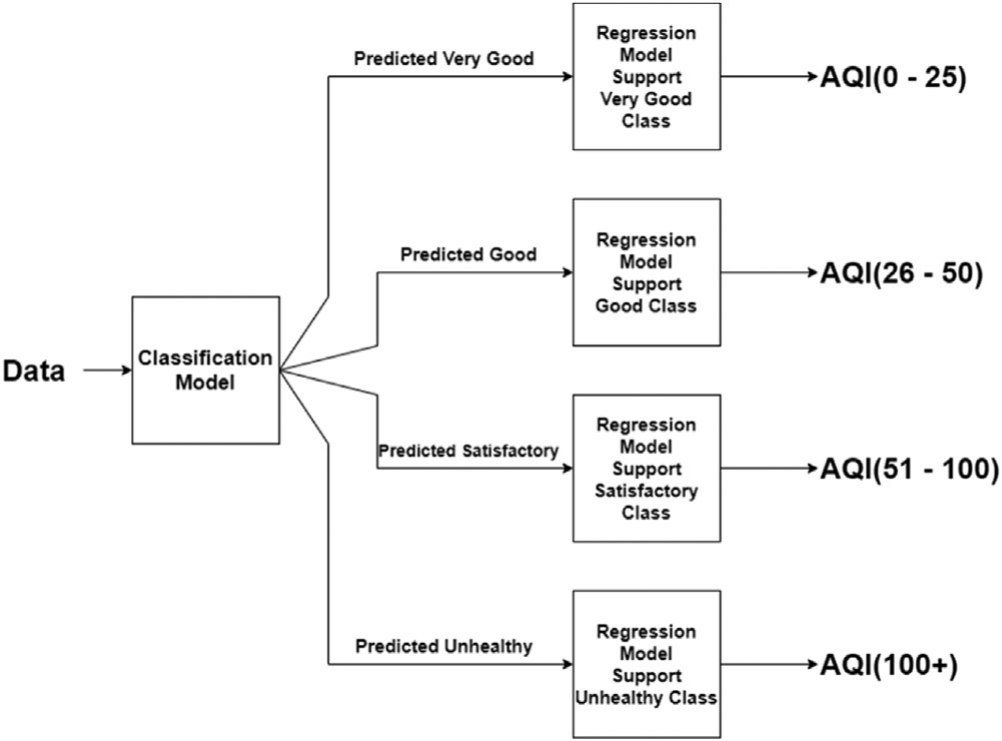
Our proposed hybrid model.

In this work, we propose and test two designs of our models. In the first design, the intuition behind comes from our previous phase [24], and a possibility that a model to correctly classify input data into an air quality standard class and a model to actually estimate the final AQI values may not be the same. So, with this design, we do one step at a time. First, we classify the input data into an AQI class, and then we estimate the final AQI values once the data points are correctly classified into that group using an appropriate regression model for each class. Therefore, the design of our hybrid model consists of a classification part followed by a regression part–one for each of the air quality standard's classes. An overview of our proposed hybrid model is shown in Fig. 2. Another design in our approach is a one-pass model, which involves only one regression model from start to finish.

All the supervised machine learning techniques mentioned in the Materials and Methods section are used in either the classification part, the regression part, or both, as outlined in that section. The reasons for selecting certain supervised machine learning techniques are two-folded: 1) based on our preliminary surveying of methods that showed positive potential, and 2) inspired by a literature review conducted during the time of research.

### Model evaluations

To evaluate the performance of our models, we divide how the models are evaluated into two ways: 1) using classification model performance metrics and 2) using regressor model performance metrics.

(1) Classification model performance metrics

For performance evaluation of the first part (classification model), we use confusion matrix to measure performance by considering accuracy, precision, recall, and F1 score. The average value from all classes is based on macro average value. Confusion matrix is calculated by counting the frequency of correctly predicted values against the actual values of each class [31]. Since we have more than 2 classes, our confusion matrix is as shown in Table 3.

• T_x_ are True Positive of class x.
• F_xy_ are False prediction—true class is x, but prediction is y.
• Example of confusion matrix calculation and model performance calculation is as follows.
• True Positive of class 1: T_1_
• True Negative of class 1: T_2_ + T_3_ + T_4_ (T_x_; x ≠ 1)
• False Positive (class 1): F_21_ + F_31_ + F_41_ (Sum of F_x1_)
• False Negative (class 1): F_12_ + F_13_ + F_14_ (Sum of F_1y_)

**Table 3:** Example of confusion matrix for more than 2 classes.

Confusion Matrix		Actual			
		1	2	3	4
Predicted	1	T_1_	F_21_	F_31_	F_41_
	2	F_12_	T_2_	F_32_	F_42_
	3	F_13_	F_23_	T_3_	F_43_
	4	F_14_	F_24_	F_34_	T_4_

The equations to calculate these metrics are as follows:

(4) $$Accuracy=\;\frac{TP_{x}\;+\;TN_{x}}{n}$$

(5) $$Precision_{x}=\;\frac{TP_{x}}{TP_{x}+\;FP_{x}}$$

(6) $$Recall_{x}=\;\frac{TP_{x}}{TP_{x}+\;FN_{x}}$$

(7) $$F1\;Score_{x}=2\frac{Precision_{x}*\;Recall_{x}}{Precision_{x}+\;Recall_{x}}$$

(2) Regressor model performance metrics

For regressor model performance metrics, we use mean absolute error (MAE) and coefficient of determination (R^2^) to measure performance of the models. The best model will be determined by evaluating MAE first (first priority) and then R^2^ (second priority), respectively. These two metrics can be calculated by the following equations:

(8) $$MAE=\;\frac{\sum_{i}^{n}\left|Observed_{i}\;-\;Predicted_{i}\right|}{n}$$

(9) $$R^{2}=1-\;\frac{\sum_{i}^{n}{\left(Observed_{i}\;-\;Predicted_{i}\right)}^{2}}{\sum_{i}^{n}{\left(Observed_{i}\;-\;\frac{\sum_{i}^{n}Observed_{i}}{n}\right)}^{2}}$$

## Data analysis, experiments, and discussions on results

In this section, we first provide an overview of the data analysis process, including the rationale behind and goals of our data analysis. We then design and conduct experiments, followed by a discussion on each experimental results. The overall steps in our data analysis can be highlighted as follows:

### Data analysis process

**Table:** Overall flow of the data analysis process.

Defining goals of our data analysis → cleaning and preparing data → performing experiments → analyzing experimental results and communicating findings

(1) Defining goals

We can divide our goals into 4 categories as follows:

(1) to identify the best configurations for the classification part of the proposed hybird model;
(2) to identify the best configuration for the regression part (which predicts the actual AQI values) of the proposed hybrid model;
(3) to identify the best configuration for the pure regression model and compare the resulting performances between the two (the proposed hybrid model and the pure regression model);
(4) to conduct additional comparison experiments with other approaches similar to our work, in order to confirm our model's performance.
(2) Cleaning and preparing data

We prepare a dataset from Landsat 8 images. Datasets used in this work must be in a valid format; within range 0 to 10,000 [17]. Additionally, only dataset with low cloud confident are selected (cloud confidence refers to how likely cloud are present in the data), that way, outlier problem can be avoided. In this phase of study, we also have more data added to our datasets. However, not all the data is high quality, and so they have some errors. We itemized each data by error type, along with how many data fall into each of the error class as described in Table 13 below.

After checking and verifying the datasets, we have selected correct/normal datasets of 982 records as shown in Table 4.

**Table 4:** Our dataset class distribution.

Class	Count
Very Good	407
Good	303
Satisfactory	155
Unhealthy	113
Very Unhealthy	4
**Total**	**982**

In this step, we also add spectral vegetation indices (VI, NDVI, and TVI) into the dataset by using Eqs. (1)–(3). Our dataset is increased from our previous paper [24]; previously we had only 390 observations). So, now we have a total of 982 datasets, covering a total of 7 bands (bands 1–7) + 3 (VI, NDVI, and TVI) spectral vegetation indices as described in Table 1 and (1), (2), (3). This dataset has more data and also consists of low cloud confident data. In the next section, we design experiments to confirm our model's performance.

(3) Performing experiments and analyzing results

In this section, we design our experiments into 4 configurations, consistent with the goals of our data analysis mentioned in the first step (defining goals) as follows:

• performing experiments with the classification part of the proposed hybrid model with our newly added/increased datasets,
• performing experiments with the regression part of the proposed hybrid model,
• performing experiments with the pure regression model (one-pass from start to finish), and
• conducting additional comparison experiments with other methods with our datasets.

The specifications of our machine are as follows:

• CPU: AMD Ryzen 5 3600 (6 cores/12 threads), 4.0 GHz
• RAM: 32GB (4 × 8GB) DDR4 with a bus speed of 2666 MHz
• GPU: Nvidia GeForce RTX 2060 with 6GB VRAM (1920 CUDA cores) (for neural networks training)

We perform these experiments with this machine specifications, discuss the experimental results, and communicate our findings next.

### Experiments

(1) Performing experiments with the classification part of the proposed hybrid model with our newly added/increased datasets

In this first experiment, following up from our previous work (in which we predicted only the class of air quality according to the Pollution Control Department, Ministry of Natural Resources and Environment of Thailand standards: Very good, Good, Satisfactory, Unhealthy, and Very Unhealthy [12]–not the actual AQI values), we re-test our model in the classfification part (the first part of the proposed hybrid model) to see if the same model and accuracy performance still holds in the classification part of the model. In our previous paper [24], we reported that Random Forest model performed the best with an averaged accuracy of 0.914, averaged precision of 0.89, averaged recall of 0.814, and an averaged F-1 score of 0.84825. However, in this work, we have more datasets, and after conducting experiments with the newly added/increased datasets (using each of the supervise machine learning techniques mentioned in Materials and Methods section with the configuration in Table 5), the experimental results are shown in Table 5. As you can see from the table, the Random Forest model no longer performs the best, and all other models also show poor performances.

**Table 5:** Classification results for this new dataset.

Model	Configuration*	Accuracy	Average Precision⁎⁎	Average Recall⁎⁎	Average F1 Score⁎⁎
Decision Tree	Criterion = entropy	0.28	0.2	0.2	0.2
K-Nearest Neighbors	*k* = 1, weights = uniform	0.33	0.28	0.27	0.27
Random forest	*n* = 114, Criterion = entropy	0.35	0.24	0.25	0.24
Gradient Boosting	*n* = 106, loss = log_los, criterion = friedman_mse	0.34	0.2	0.23	0.21

⁎ Configurations are from Nattadet et al. [24].

⁎⁎ Average values are macro average values.

These inconsistent and poor performances lead us to the next question regarding the datasets themselves. We then take a closer look at the context surrounding the data to see if there is any other factor that could potentially affect the model's performance. Then, we noticed that air quality in Bangkok is usually poor during the winter months (November - January) of each year—which is the ‘time’ factor. Therefore, we decided to incorporate this fact into our model as well. We added a ‘day of year’ (DOY) field to represent the data collection date, as air pollution in Thailand is highly affected by the time of the year. This may play an important role, and we would like to test it.

The goal of the next experiment is to test whether DOY plays an important role in this. However, before doing so, we also identified another problem that this new dataset has: the scarcity of data in one of the classes, “Very Unhealthy” class, in which we have only 4 out of 982 observations as mentioned in Table 4. It is very likely that these few data points will be accidently treated as noise by the model, if left as-is. So, we decided to combine data from the “Very Unhealthy” class with the “Unhealthy” class together to form a new class; the range of AQI values for this class is also expanded as a result. We, then, conducted the experiments with this new class with DOY included, and the results are shown in Table 6; as we shall see that the overall performance of all models has also improved (Tables 5 and 6).

**Table 6:** Classification results with “DOY” field added.

Model	Configuration*	Accuracy	Average Precision⁎⁎	Average Recall⁎⁎	Average F1 Score⁎⁎
Decision Tree	Criterion = entropy	0.69	0.7	0.7	0.7
**K-Nearest Neighbors**	***k*=1, weights = uniform**	**0.8**	**0.79**	**0.81**	**0.8**
Random forest	*n* = 114, Criterion = entropy	0.7	0.7	0.68	0.68
Gradient Boosting	*n* = 106, loss = log_los, criterion = friedman_mse	0.74	0.78	0.76	0.77

⁎ Configurations are from Nattadet et al. [24].

⁎⁎ Average values are macro average values.

*Discussions on results:* This time, from the results, the overall performances of all the models have improved, and we found that the best model has changed from Random Forest model in our previous paper (in our previous paper, the conclusion was that the best model was random forest with *n* = 114 and criterion = entropy, with accuracy = 0.914, average precision = 0.89, average recall = 0.814 and average F1 score 0.84285 - all average values are macro average values.) to k-nearest neighbors model in this experiments with *k* = 1, which achieved an accuracy = 0.8, an average precision = 0.79, an average recall = 0.81 and an average F1 score 0.8.

(2) Performing experiments with the regression part of the proposed hybrid model

In this next experiments, we would like to continue with the second part (regression part) of the proposed hybrid model—this regression part is responsible for estimating the final AQI values. Our initial hypothesis was that a regressor model for each class may not necessary to be the same one for all the classes (one size does not need to fit all). So, we design the experiments to identify the best regressor model for each of the classes to predict the actual AQI values as shown in Fig. 2.

**Fig. 3:**
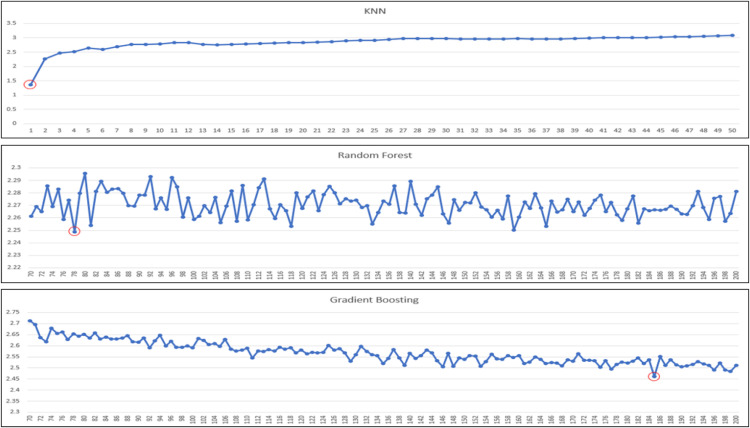
The performance of “Very Good” predicted class using KNN and ensembles based on MAE.

**Fig. 4:**
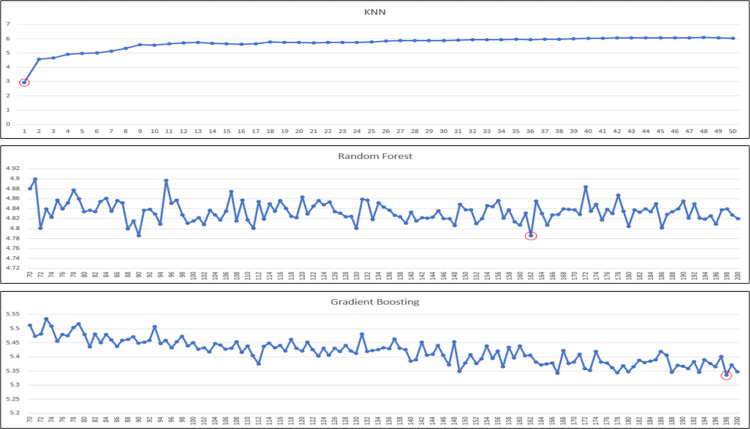
The performance of “Good” predicted class using KNN and ensembles based on MAE.

**Fig. 5:**
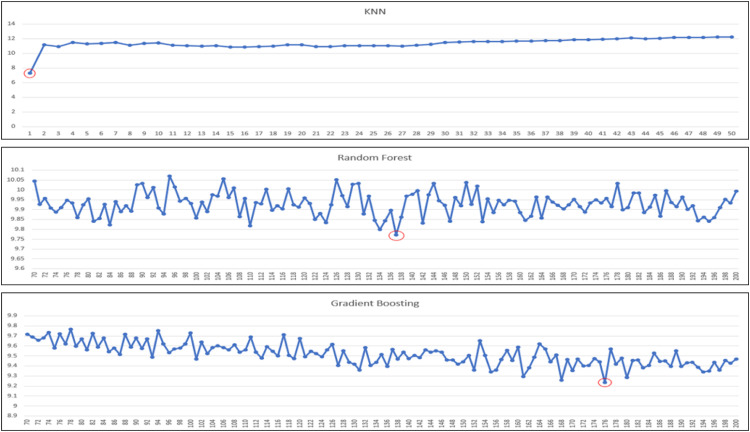
The performance of “Satisfactory” predicted class using KNN and ensembles based on MAE.

Our regressor models to experiment/test are: linear regression, decision tree (regressor), k-nearest neighbors (regressor), random forest (regressor) and gradient boosting (regressor). In the case of k-nearest neighbors and ensemble techniques (i.e., random forest and gradient boosting), there are also further hyperparameters to consider: ‘k’ and ‘n’. For KNN, it is ‘k’ and for ensembles, it is ‘n’, in which ‘k’ means how many neighbors of the target data is considered for predictions, and ‘n’ means the number of estimators used in ensemble techniques. Because the performances of models are impacted by these parameters, we need to tune these two parameters also. We further tune models to identify the best ‘k’ or ‘n’ estimators that yield highest performance. The ranges of ‘k’ and ‘n’ estimators for experiments are shown in Table 7 (Fig. 3, Fig. 4, Fig. 5).

**Table 7:** Range of k or n estimators tuned for corresponding regressor model.

Model	Range of k or n estimators
K-Nearest Neighbors	1 - 20
Random forest	70 - 200
Gradient Boosting	70 - 200

**Fig. 6:**
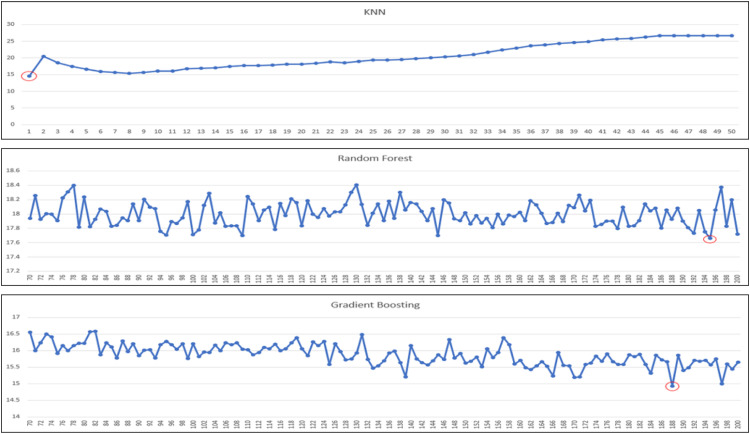
The performance of “Unhealthy” predicted class using KNN and ensembles based on MAE.

For the best k or n estimator selection, we will call it the best configuration for the model if it gives the best performance in terms of mean absolute error (minimum MAE). In the following, we perform a series of experiments for all the aforementioned regressor models, and for KNN and ensemble techniques, we further experiment with a range of k or n estimators to determine the best optimal performances. A series of graphs of k (for KNN) or n estimators (for random forest or gradient boosting) tuning to identify the best of k or n estimators for each predicted class is shown in Fig. 3, Fig. 4, Fig. 5, Fig. 6, with the best optimized hyperparameters red circled in the graphs. The final results of best k or n estimators for each predicted class are summarized in Table 8.

**Table 8:** Results of KNN and ensemble models tuning.

Predicted Class	Model	Best k or n estimators
“Very Good” Class	K-Nearest Neighbors	*k* = 1, weights = uniform
	Random forest	Estimators = 78, Criterion = MAE
	Gradient Boosting	Estimators = 185, Loss = MAE
“Good” Class	K-Nearest Neighbors	*k* = 1, weights = uniform
	Random forest	Estimators = 162, Criterion = MAE
	Gradient Boosting	Estimators = 198, Loss = MAE
“Satisfactory” Class	K-Nearest Neighbors	*k* = 1, weights = uniform
	Random forest	Estimators = 137, Criterion = MAE
	Gradient Boosting	Estimators = 176, Loss = MAE
“Unhealthy” Class	K-Nearest Neighbors	*k* = 1, weights = uniform
	Random forest	Estimators = 195, Criterion = MAE
	Gradient Boosting	Estimators = 188, Loss = MAE

After we get the best configurations for KNN, random forest and gradient boosting, we compare the results of all regressor models to identify the best model of each predicted class. The result of each model will consider MAE first. The regressor model configuration for each predicted class are shown in Table 9 and the respective regressor model performance comparison is shown in Table 10.

**Table 9:** Configurations of each regressor model for each predicted class.

Predicted Class	Model	Configuration
“Very Good”	Linear Regression	–
	Decision Tree	Criterion = MAE
	K-Nearest Neighbors	*k* = 1, weights = uniform
	Random forest	Estimators = 78, Criterion = MAE
	Gradient Boosting	Estimators = 185, Loss = MAE
“Good”	Linear Regression	–
	Decision Tree	Criterion = MAE
	K-Nearest Neighbors	*k* = 1, weights = uniform
	Random forest	Estimators = 162, Criterion = MAE
	Gradient Boosting	Estimators = 198, Loss = MAE
“Satisfactory”	Linear Regression	–
	Decision Tree	Criterion = MAE
	K-Nearest Neighbors	*k* = 1, weights = uniform
	Random forest	Estimators = 137, Criterion = MAE
	Gradient Boosting	Estimators = 176, Loss = MAE
“Unhealthy”	Linear Regression	–
	Decision Tree	Criterion = MAE
	K-Nearest Neighbors	*k* = 1, weights = uniform
	Random forest	Estimators = 195, Criterion = MAE
	Gradient Boosting	Estimators = 188, Loss = MAE

**Table 10:** Performance results of each regressor model for each predicted class.

Predicted Class	Model	MAE	R^2^
“Very Good”	Linear Regression	3.724812827	−0.007804389
	Decision Tree	1.696	0.479183808
	K-Nearest Neighbors	1.36	0.586967254
	Random forest	2.211179487	0.569412096
	Gradient Boosting	2.33029211	0.454437082
“Good”	Linear Regression	5.870447831	0.011584563
	Decision Tree	4.674418605	0.09720783
	K-Nearest Neighbors	2.918604651	0.363667765
	Random forest	4.722437554	0.305527453
	Gradient Boosting	5.174536175	0.123705025
“Satisfactory”	Linear Regression	12.01142116	−0.033751627
	Decision Tree	11.19565217	−0.477303958
	K-Nearest Neighbors	7.326086957	0.023221455
	Random forest	9.558235481	0.308781089
	Gradient Boosting	8.713142288	0.412421994
“Unhealthy”	Linear Regression	28.30169912	−0.239189192
	Decision Tree	12.55263158	0.392962918
	K-Nearest Neighbors	15.98245614	0.445747726
	Random forest	17.24520918	0.454456633
	Gradient Boosting	13.87447744	0.592495801

**Fig. 7:**
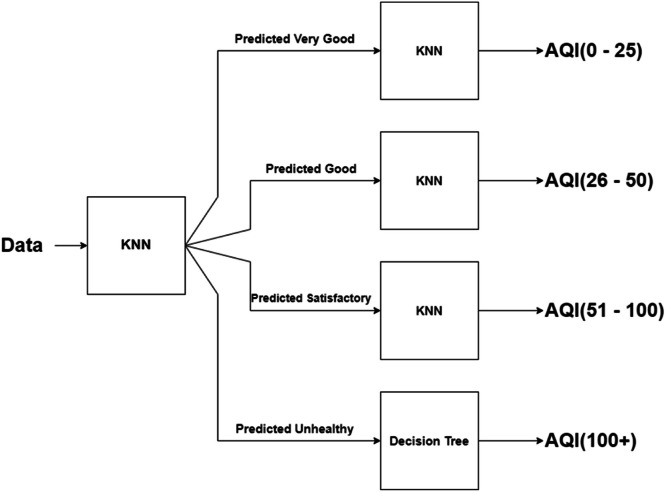
Our hybrid model with selected regressors.

From our experimental results shown in Table 10, for the “Very Good” predicted class, KNN is the best model with MAE = 1.36 and R^2^ = 0.587. For the “Good” predicted class, KNN is the best model with MAE = 2.9186 and R^2^ = 0.3637. For the “Satisfactory” predicted class, KNN is the best model with MAE = 7.3261 and R^2^ = 0.0232. And for the “Unhealthy” predicted class, decision tree is the best model with MAE = 12.5526 and R^2^ = 0.393. The resulting selected best regressors of our hybrid model are summarized in Fig. 7.

*Discussions on results:* From the hybrid model (Fig. 7), we calculate the performance for each part and the overall part as follows: for the classification part, the results are as follows: Accuracy = 0.8, Average Precision = 0.79, Average Recall = 0.81 and Average F1 Score 0.8—all average values are Macro Average Values. And, for the regression part, we get: MAE = 4.3567 and R^2^ = 0.9468 (calculated as if the classification part is 100% correct). Therefore, the overall model performance is MAE = 8.3864 and R^2^ = 0.7499, as shown in Fig. 9.

(3) Performing experiments with the pure regression model (one-pass from start to finish)

**Fig. 8:**
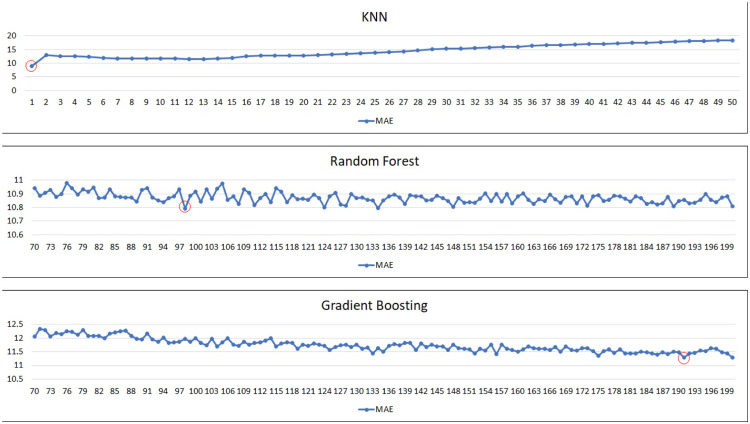
The performance of KNN and ensembles tuned in pure regressor model based on MAE.

**Fig. 9:**
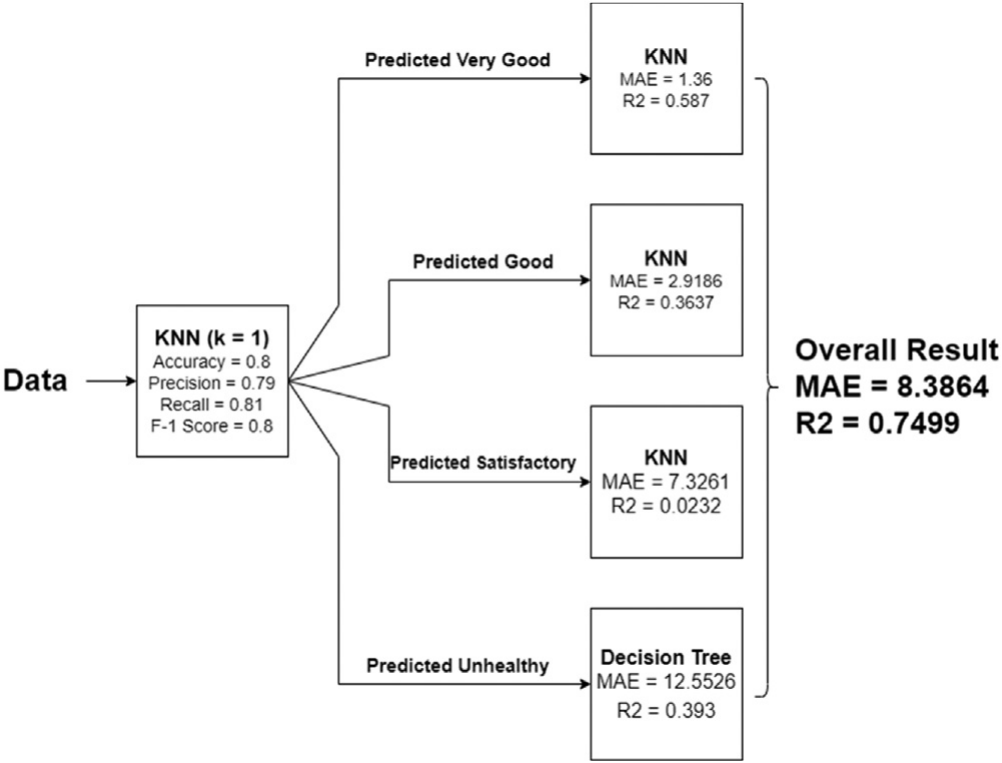
Hybrid model overall performance results.

**Fig. 10:**
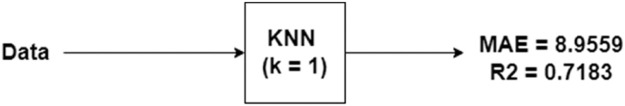
Pure regressor model performance results.

**Fig. 11:**
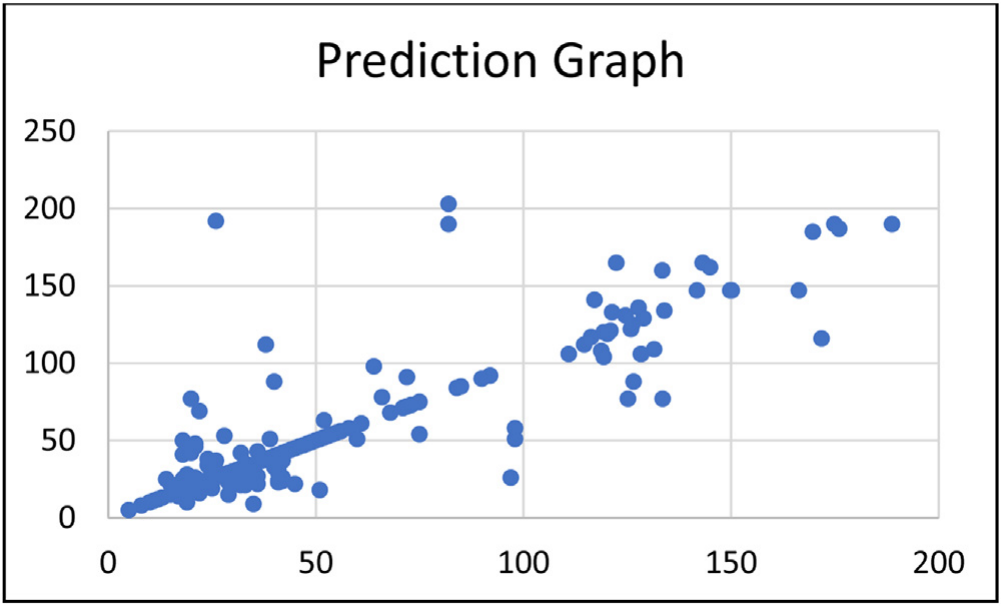
Hybrid model prediction graph.

**Fig. 12:**
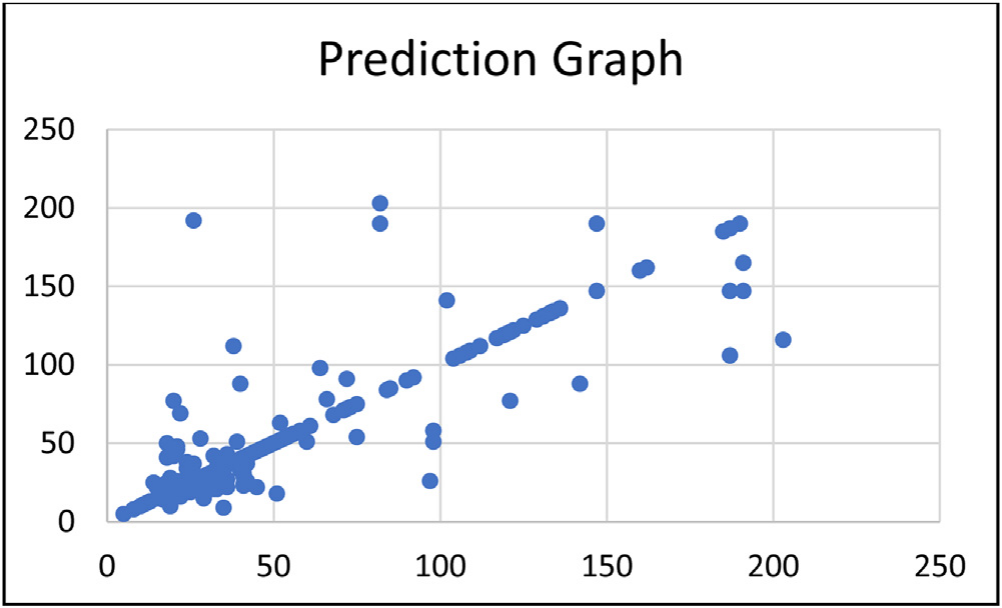
Pure regressor model prediction graph.

**Fig. 13:**
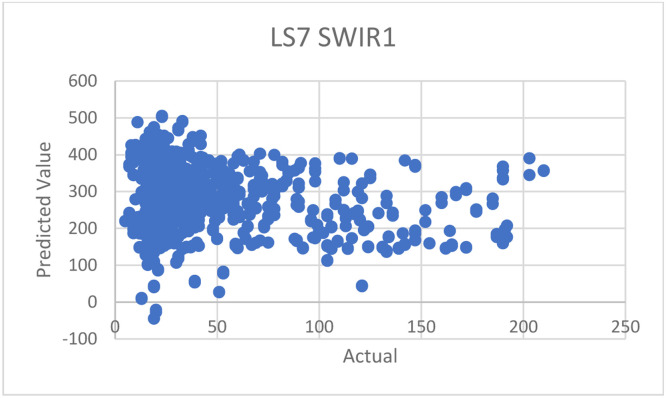
Prediction graph for the approach by C. Mozumder et al. [8]—with SWIR1 band data.

**Fig. 14:**
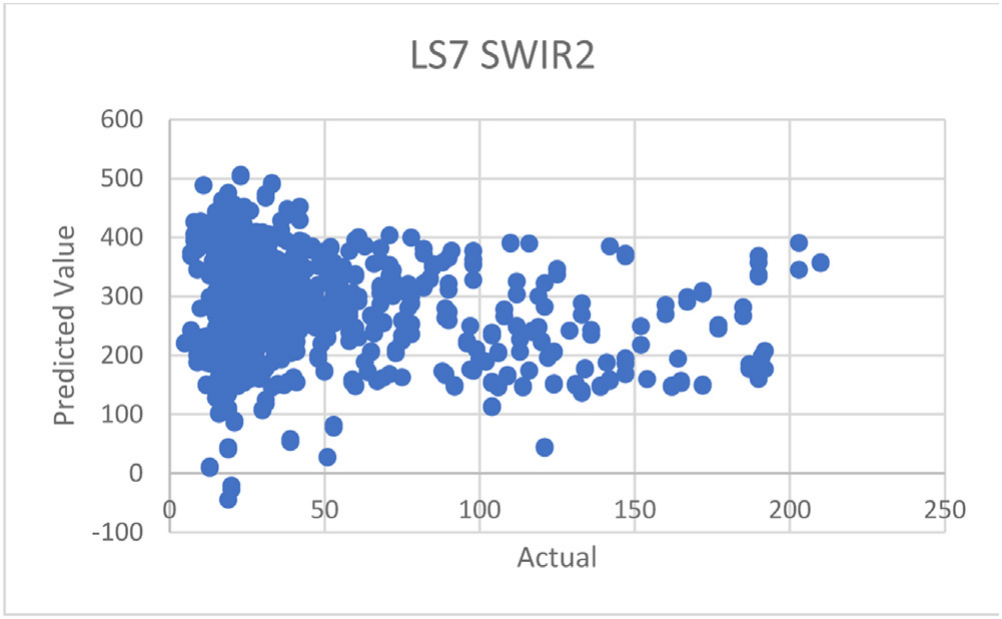
Prediction graph for the approach by C. Mozumder et al. [8]—with SWIR2 band data.

**Fig. 15:**
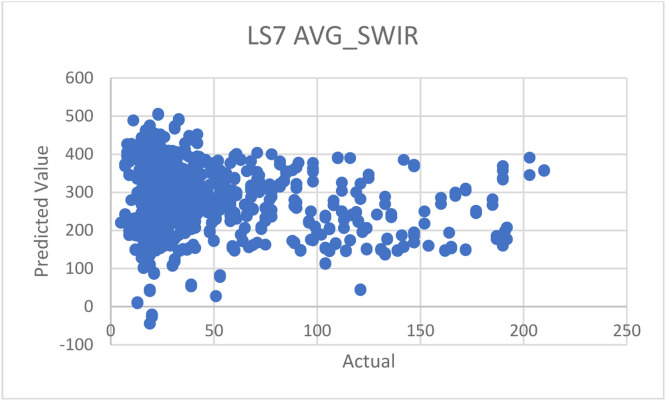
Prediction graph for the approach by C. Mozumder et al. [8]—with averaged SWIR1 and SWIR2 band data.

**Fig. 16:**
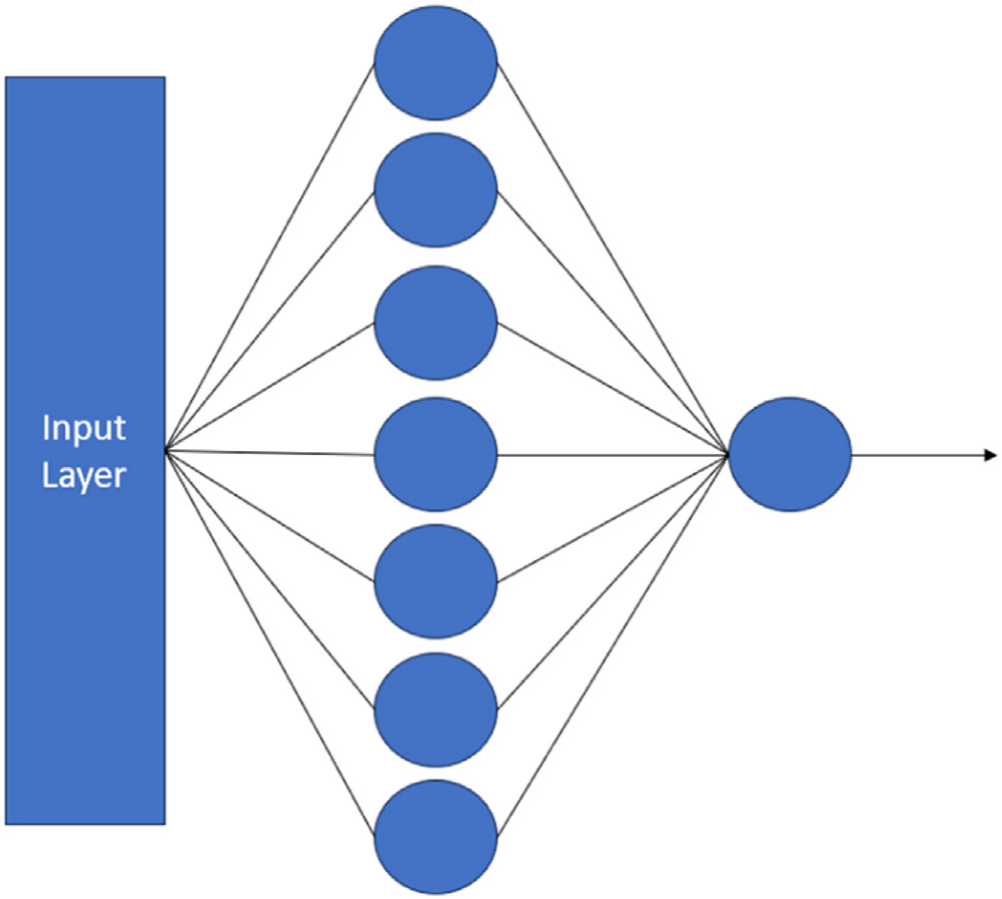
Structure of Spatial Back Propagation Neural Network Model in the paper.

**Fig. 17:**
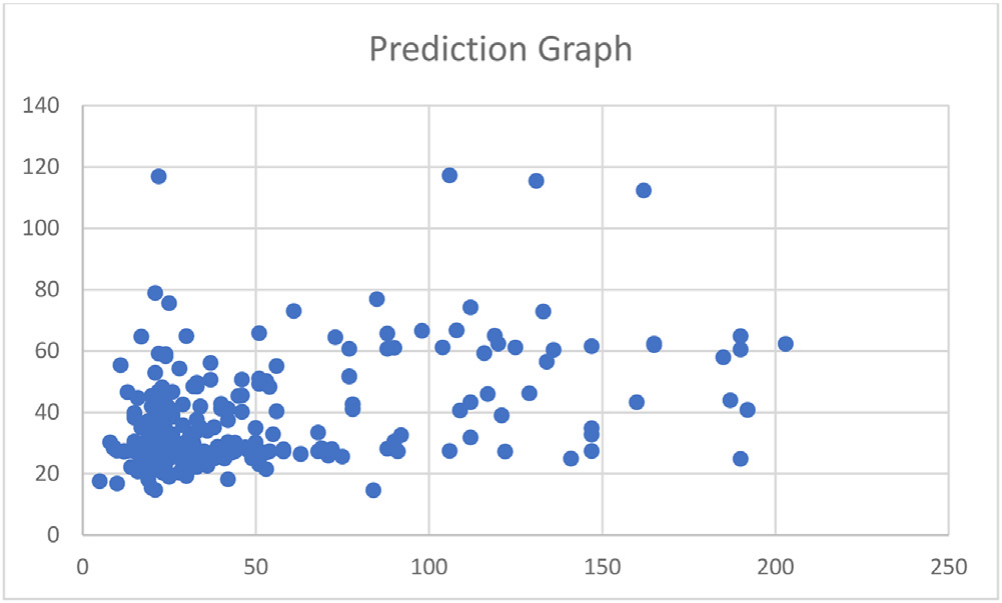
Prediction graph for the approach by Wang, et al. [13].

After we identified the best configuration for the hybrid model in the previous experiments, next, we would also like to experiment with pure regressor models as well – using only one regression model from start to finish in predicting actual AQI values. And then, comparing them with the hybrid model. For configurations of KNN or ensembles for pure regressor models, we tune them in the same fashion as we do for the regressor part of the hybrid model. The results of parameter tuning are shown in Fig. 8, and the best configuration for each regressor model is also shown in Table 11, also red-circled in the graphs.

**Table 11:** Configurations of each regressor model for pure regressor model.

Model	Configuration
Linear Regression	–
Decision Tree	Criterion = MAE
K-Nearest Neighbors	*k* = 1, weights = uniform
Random forest	Estimators = 98, Criterion = MAE
Gradient Boosting	Estimators = 191, Loss = MAE

The overall performance results of each model are shown in Table 12. For the results of pure regression techniques when considering MAE first, KNN gives the lowest (the best) performance among other models.

**Table 12:** Performance results for pure regressor model.

Model	MAE	R^2^
Linear Regression	28.1998383	0.20500946
Decision Tree	12.40677966	0.634164936
K-Nearest Neighbors	8.955932203	0.718283228
Random forest	10.59320304	0.832397213
Gradient Boosting	10.808192	0.813546129

**Table 13:** Itemized collected data by error types.

Type of error in data	Count
Missing interpolated data	76
False saturation data (with values ranging less than 0 or greater than 10,000)	8
High or middle cloud confidence data	182
Normal data	982
**Total collected data**	**1248**

For pure regression model approach. we get the overall performance of pure regressor model with MAE = 8.9559 and R2 = 0.7183 as shown in Fig. 10.

*Discusssions on results:* From Figs. 11 and 12, we can see that both graphs are quite similar. When considering both prediction graphs as well as the overall performance metrics, we conclude that the hybrid model has a slightly better performance than the pure regressor model—but both models can fit with our dataset (with R^2^ > 0.7 for both models).

(4) Performing additional comparison experiments with other methods with our datasets.

To further confirm the performance of our model, we conducted comparison experiments with other approaches similar to ours. Out of 12 works as mentioned in Table 15, there are three approaches similar to what we are doing: 1) an approach by C. Mozumder et al. [8], 2) another approach by W. Wang, et al. [13], and 3) an approach by Q. Di, et al. [9].

*To compare the performance with an approach by Mozumder* et al. [8]*:* we used the suggested configurations and equations provided by the paper with our datasets in Thailand—although the original datasets applied in the paper was Landsat 7, which is not exactly the same as ours, which is Landsat 8 but still in the same series of landsat imagery. The suggested equation is as follows:

AQI=851.6TVI−6.4VI+NIR−10.4SWIR−460

From the equation above, we encountered one issue related to the SWIR data, specifically. This is because Landsat 7 satellite (used in the paper) has only one SWIR band, while our data of Landsat 8 has two SWIR bands (SWIR1 and SWIR2), as mentioned in Table 1. To address this issue, we conducted experiments in 3 ways: 1) using only SWIR1, 2) using only SWIR2, and 3) using the average data between SWIR1 and SWIR2. The results of these experiments are shown in the following Table 14.

**Table 14:** Experimental results with our dataset, compared to other approaches.

Dataset	R^2^	MAE
Mozumder et al. [8]—with SWIR1 band data only	−35.0055	226.0215
Mozumder et al. [8]—with SWIR2 band data only	−35.1707	226.6137
Mozumder et al. [8]—with averaged SWIR1 and SWIR2 band data	−35.0880	226.3176
Wang, et al. [13]	0.1062	24.1021
Pure regression model	0.7183	8.9559
**Hybrid model**	**0.7499**	**8.3864**

**Table 15:** Summary of literature review of each paper.

Article	Year	Datasets	Methods	Advantages	Disadvantages	Study area
[8] C. Mozumder, K.V. Reddy, and D. Pratap, Air pollution modeling from remotely sensed data using regression techniques, Journal of the Indian Society of Remote Sensing 41 (2013): 269–277.	2012	– Landsat 7 data – IRS data	– Linear regression	– Used few data sources – Provided good details of every other data used in the paper	– Prediction methods employed are too few – Lack of comparison experiments with other methods – The provided model does not work well with other satellite data	India
[9] Q. Di, et al., Assessing PM2. 5 exposures with high spatiotemporal resolution across the continental United States, Environmental science & technology 50.9 (2016): 4712–4721.	2016	– Satellite Aerosol Optical Depth data (AOD) – Land use data	– Convolutional neural networks	– Utilized satellite images as one of the data sources – Applied convolutional neural networks to satellite datasets	– Did not give enough details of hyperparameters, needed for the method to be re-implemented and reproduced.	USA
[10] H.S. Pannu, D. Singh, and A.K. Malhi, Multi-objective particle swarm optimization-based adaptive neuro-fuzzy inference system for benzene monitoring, Neural computing and applications 31 (2019): 2195–2205.	2017	– Multi-source data	– PSO (particle swarm optimization)	– Innovatively used particle swarm optimization (PSO) methods	– Focused on benzene rather than PM_2.5_ or PM_10_ – Not enough explanation about the dataset “MAPAN”	India
[11] M. Zamani Joharestani, et al., PM2. 5 prediction based on random forest, XGBoost, and deep learning using multisource remote sensing data, Atmosphere 10.7 (2019): 373.	2019	– Satellite Aerosol Optical Depth data (AOD) – Meteorological data	– Random Forest – Extreme Gradient Boosting – Deep Learning	– Used many methods and compared the results, where the neural networks method appears the be the best	– Lack of necessary details of all models’ hyperparameters	Tehran, Iran
[12] J.K. Sethi, and M. Mittal, Ambient air quality estimation using supervised learning techniques, EAI Endorsed Transactions on Scalable Information Systems 6.22 (2019): e8-e8.	2019	– Meteorological data	– Decision trees – SVM – Naïve Bayes – Random forest – Voting ensemble – Stacking ensemble	– Used many techniques, and conducted comparison experiments to identify the best methods – Utilized both classification and regression techniques	– Didn't provide enough details of hyperparameters of all the models	China
[13] W. Wang, et al., Estimation of PM2.5 concentrations in China using a spatial back propagation neural network, Scientific reports 9.1 (2019): 13,788.	2019	– MODIS – AOD – Meteorological data	– Neural networks	– Applied neural networks and provided some information about hyperparameters, as well as how and why they were tuned that way – Used satellite data of “MODIS”	– Lack of details required for re-implementation and reproduction, such as how much epochs used and what activations of output layers are	China
[14] X. Sun, et al., Dynamic monitoring of haze pollution using satellite remote sensing, IEEE Sensors Journal 20.20 (2019): 11,802–11,811.	2020	– MODIS	– V5.2 algorithm	– Showed clear step by step to prepare data – Used satellite data from “MODIS”	– Lack of an explanation about V5.2 algorithm	Shanghai, China
[15] W.C. Leong, R.O. Kelani, and Z. Ahmad, Prediction of air pollution index (API) using support vector machine (SVM), Journal of Environmental Chemical Engineering 8.3 (2020): 103,208.	2020	– Meteorological data	– SVM	– Provided a flowchart of the method, making it easy to understand	– Lack of an explanation about the datasets – The organization of sections and sections are hard to follow	Perak and Penang States, Malaysia
[16] Y. Gu, B. Li, and Q. Meng, Hybrid interpretable predictive machine learning model for air pollution prediction, Neurocomputing 468 (2022): 123–136.	2022	– Air pollution datasets	– HIP-ML (Hybrid Interpretable Predictive Machine Learning)	– Proposed a new approach of machine learning or deep learning	– The organization of paper is really hard to follow	China
[17] F. Lin, C. Gao, and K.D. Yamada, An Effective Convolutional Neural Network for Visualized Understanding Transboundary Air Pollution Based on Himawari-8 Satellite Images, IEEE Geoscience and Remote Sensing Letters 19 (2021): 1–5.	2022	– Himawari-8 data (Meteorological Satellite)	– Convolutional neural networks	– Employed many CNN models to compare the predictions	– Resulting tables are not easy to read and understand – Lack of the full details of models’ performance results	North–East Asia region
[18] H. Ji, et al., Research on adaption to air pollution in Chinese cities: Evidence from social media-based health sensing, Environmental research 210 (2022): 112,762.	2022	– Weibo health data – Meteorological data	– Generalized additive model (GAM)	– Utilized also data from social networks that is rapidly growing, help stabilizing the model	– Weibo data mentioned in the paper did not cover the entire China – The reliability of social media data is questionable, and lack of data filtering process	Urban of China
[19] M. Saez, and M.A. Barceló, Spatial prediction of air pollution levels using a hierarchical Bayesian spatiotemporal model in Catalonia, Spain, Environmental Modelling & Software 151 (2022): 105,369.	2022	– Air pollution data	– Hierarchical Bayesian	– Provided a very clear details of data description, i.e., what type of data is reported from which stations, and how many stations report a particular data – Models perform well with O_3_ and NO_2_ predictions	– Resulting tables are inconsistent and not easy to follow. – Models perform poorly when predicting PM_2.5_ or PM_10_	Catalonia, Spain

And then, we also created a prediction plot for each experiment, as we did in the previous experiment section. If the model fits the data, the data plot should be in a diagonal pattern on the graph. When plotting the graphs similar to Figs. 11 and 12, the resulting plots are shown in Fig. 13, Fig. 14, Fig. 15. As you can see, there are mostly scattered—no diagonal, indicating poor performances in predictions.

*Discussions on results:* From the experiment results and plots, we can see that the proposed method by C. Mozumder et al. (2012)’s paper were not suitable to Thiland and consequently lead to the poor performances, compared to ours. We think that this is as a result of the datasets used in the approach being different and not suitable for Thailand. Moreover, the equation provided in the paper is formulated based on Landsat 7, which may not be fully compatible with Landsat 8, even though they belong the same series; for example, Landsat 7 has only one SWIR band, whereas Landsat 8 has two SWIR bands, as mentioned previously. Additionally, this provided equation was derived by considering context of data in India—not Thailand. Finally, it is also possible that the mathematic model alone may not be sufficient in estimating the actual AQI values, when compared to machine learning approaches.

*To compare the performance with an approach by Wang* et al. [13]*:* For this second approach by W. Wang et al. (2019), the structure of spatial back propagation neural network model employed in the paper is shown in Fig. 16. However, not all parameters were specified in the paper. So, we experimented this model with our dataset by tuning batch_size = 32 and epochs = 15,000, and we got the model's performance results as follows: MAE = 24.1021 and R2 = 0.1062, as shown in Table 14. In addition, we also plotted the prediction graph (as shown in Fig. 17) to see if model's performance will fit the diagonal pattern in the graph.

From the prediction graph (as shown in Fig. 17), because the prediction graph does not fit in the diagonal pattern. So, we conclude that model also does not perform well with our dataset.

*To compare the performance with an approach by Di* et al. [9]*,* the approach by the paper also has a similar method to us. However, it will not work with our datasets. This is because the details as to what the configurations is used, and how the structure of artificial neural networks looks like are missing. Thus, we do not have enough details in order to re-implement and compare the experiments fairly. So in conclusion, we can say that our work, compared to other similar approaches, can peform the complete steps as promised (from identifying the class of AQI to estimating the actual AQI values) with good results/performances.

### Main limitations and lessons learned

One of the main limitations of this work is that the satellite data we used to build our approach is from Landsat 8. As a result, it is possible that our approach might not work as anticipated with imagery from other satellites. This could be due to differences in band data and other attributes or properties, such as sensors and technologies used. Therefore, we recommend thoroughly assessing the compatibility of our approach with data from other satellites first. If it is not compatible, follow our steps and processes to derive the right methodology for the specific satellite data.

Another limitation comes from the fact that the way our data is reported by the Pollution Control Department of Thailand differs from others—the current reported data is calculated based on the average of values from the past 24 h combined with the current value. So, when applying our method to a study area where the nature of current value calculation is different from ours, we may get unexpected results.

In terms of lessons learned from this study, particularly for those interested in applying our research to their work, as in our study, we identified one specific factor, the day of the year (DOY), which greatly contributes to the model's accuracy due to the seasonal variation in air quality in the study area, which are Bangkok and its surrounding metroplex. Therefore, we recommend that other researchers identify potential factors that might play an important role in estimating AQI values in their specific study areas, and then test and incorporate them into their models, if appropriate.

## Conclusions and future work

In this work, we propose models to estimate AQI values from satellite images. The models help eliminating the situations where physical air quality assessment devices are limited and scare, especially in the developing/emerging countries by utilizing satellite imagery and machine learning techniques. We propose and compare two approaches: a hybrid model (where the estimation is divided in 2 part: a classification of AQI classes and then a regression of estimating the actual final AQI values), and a purely regressor model (a traditional approach where the model directly estimates the AQI values from inputs). In this work, we have added more data into our datasets. We have also added and tested another potential factor, the day of year (DOY), that has effects on the performances of our models. The results of adding this factor show a positive impact on the performance of our models, as seen in the increased accuracy results. For performance comparison, we focus on mean absolute error (MAE) for each model as the first priority. We then consider coefficient of determination (R^2^) as the second priority.

As mentioned, the hybrid model is a combination of classification techniques and regression techniques together. In this work, for classification part in the hybrid model, the best model is k-nearest neighbors (KNN) when *k* = 1 with performance accuracy = 0.8, average precision = 0.79, average recall = 0.81 and average F1 score 0.8 to classify data into each class according to the air quality index standard. For regression part, the classified/predicted data is sent to the respective regressor model for each class to do the final estimation of AQI. In the hybrid model, we have 4 regressor models; one for each of the 4 classes. The performance in regression part is follows: MAE = 4.3567 and R^2^ = 0.9468. Thus, the overall hybrid model performance is: MAE = 8.3864 and R^2^ = 0.7499 (Fig. 9.).

Next, we experimented with the traditional approach (which uses only regression techniques) in order to compare the performance with the hybrid approach. K-nearest neighbors (KNN) when *k* = 1 with performance with MAE = 8.9559 and R^2^ = 0.7183 yields the best performance model.

When comparing both approaches, we conclude that the hybrid model has a slightly better performance than the pure regressor models. In the hybrid model, if we consider only in the regression part, the performance is higher (less MAE, high R^2^) than the traditional approach, however when we combine the performance of classification part and regression part together, the overall performance of the hybrid model is decreased in both MAE and R^2^, but still better than the traditional approach performance as a whole. We think that the classification error has caused a significant negative impact on the overall performance of the model. In addition, we also perform additional comparison experiments with approaches similar to ours. The results also show that our approach has a better performance than theirs.

For future work, if we can find a better technique that can improve the performance of the classification part, it can help improve the overall performance. In addition, as we described earlier, we have a quality of data issue in our dataset, if we can find a better way to handle damaged data – data that has high cloud confident or data that gets false saturation, we can also increase the performance of the model accordingly as well. Lastly, for those interested in applying our methodology, we encourage to explore and test additional factors that may be location-specific to a study area or location, similar to the DOY in our case. These factors can have the potential impacts in enhancing the overall accuracy of the model as well.

## Ethics statements

Authors declare to comply with the Journal ethical guidelines.

## CRediT authorship contribution statement

**Kulsawasd Jitkajornwanich:** Conceptualization, Methodology, Investigation, Writing – original draft, Writing – review & editing, Formal analysis, Supervision. **Nattadet Vijaranakul:** Software, Investigation, Data curation, Validation, Writing – original draft, Visualization. **Saichon Jaiyen:** Validation, Formal analysis. **Panu Srestasathiern:** Validation, Formal analysis. **Siam Lawawirojwong:** Resources.

## Declaration of competing interest

The authors declare that they have no known competing financial interests or personal relationships that could have appeared to influence the work reported in this paper.

## Data Availability

Data will be made available on request.
